# Evaluation of fracture resistance and surface characteristics in monolithic zirconia: a comparative analysis of 3D printing and milling techniques

**DOI:** 10.1186/s12903-025-06570-6

**Published:** 2025-07-24

**Authors:** Rogina M. Hassan, Yomna Ibrahim, Rewaa G. AboELHassan, Amir Shoukry Azer

**Affiliations:** 1 Conservative Dentistry Department, Faculty of Dentistry, Alexandria University, Alexandria, Egypt; 2 Dental Biomaterials Department, Faculty of Dentistry, Alexandria University, Alexandria, Egypt

**Keywords:** 3D printed zirconia, Additive manufacturing, CAD-CAM, Monolithic zirconia, Subtractive manufacturing, Thermocycling

## Abstract

**Background:**

The primary method for fabrication of zirconia restorations is subtractive manufacturing technology. This process mills restorations from large blocks using various cutting tools resulting in large amounts of waste material. 3D printing has emerged as an alternative tool for additive manufacturing of zirconia with less waste and high efficiency.

**Methods:**

A total of 24 monolithic zirconia crowns were divided into: Group I (milled zirconia crowns) and Group II (3D printed zirconia crowns) (*n* = 12). The crowns were then polished and glazed then subjected to 5000 thermocycles. Fracture resistance for the crowns was measured using universal testing machine followed by estimation of Weibull modulus and characteristic strength. Fractographic analysis was done using scanning electron microscope (SEM). 72 discs (10 mm × 2 mm) were fabricated by milling and printing (*n* = 36) then subjected to 5000 thermocycles. The discs were used for surface roughness assessment both before (*n* = 12) and after (*n* = 12) glazing using contact profilometer and unglazed discs (*n* = 12) were used for microhardness which was measured by Vickers microhardness tester. Comparisons between study groups were performed using independent samples t-test. Two-way ANOVA was performed to assess the association between material (milled or printed) and glazing (glazed or unglazed) with surface roughness. Significance level was set at *P*-value < 0.05.

**Results:**

In comparison to 3D printed zirconia, the milled version exhibited comparable fracture resistance, reduced surface roughness, and increased microhardness. While both groups showed comparable fracture resistance with no significant difference (*P* = 0.26), the milled zirconia demonstrated significantly better surface finish (*P* < 0.001) and microhardness (*P* < 0.001). However, glazing lowered the surface roughness significantly for both milled (*P* < 0.001) and printed (*P* = 0.001) zirconia, bridging the gap in surface quality between the two fabrication techniques.

**Conclusions:**

The enhanced fracture resistance and Weibull modulus of 3D printed zirconia indicate increased reliability and consistency in its mechanical properties. However, limitations of its surface properties highlight the need for further optimization before full clinical adoption.

## Background

Zirconia ceramics are favored as aesthetically pleasing materials in restorative dentistry. By modifying factors such as the sintering temperature and adjusting the alumina and yttria content, both the mechanical and aesthetic properties of zirconia materials can be altered, allowing for the clinical application of monolithic zirconia restorations [1].

Yttria partially stabilized zirconia (Y-TZP) ceramics are commonly utilized due to their appealing aesthetics, biocompatibility, and superior fracture strength and toughness compared to other dental ceramics [2]. The widely utilized 3 mol% Y-TZP has demonstrated adequate mechanical properties to endure occlusal forces in posterior crowns, while the more translucent 4 and 5 mol% yttria variants are suggested for use in anterior restorations [3–5]. However, while the aesthetic qualities can be enhanced by increasing the yttria content, this comes with a decrease in fracture strength.

The primary method for creating zirconia restorations is subtractive manufacturing technology, which involves computer-aided design and computer-aided manufacturing (CAD-CAM). This process mills restorations from large blocks using various cutting tools, resulting in about 90% of the original block being discarded as waste [6]. In contrast, additive manufacturing (AM), also known as three-dimensional (3D) printing, offers a more efficient alternative with significantly less material waste as well as avoiding the high expenses associated with replacing the worn cutting tools in the subtractive manufacturing technique [7].

There are several technologies in 3D printing, including binder jetting (BJ), stereolithography (SLA), selective laser sintering (SLS) and digital light processing (DLP) [8]. Among these, SLA and DLP are regarded as the most promising methods for additively manufacturing ceramic restorations, as they allow the fabrication of ceramic parts with complex designs, fine finishing and high spatial resolution [9, 10].

In comparison to the printing of resin materials and wax patterns, the printing of dental ceramics is considered more complex. This process involves using resin as a secondary binding agent along with various other components to create a printable ceramic slurry. The 3D printing of dental ceramics is a highly intricate procedure governed by numerous factors, including initial material composition, solid loading, printing speed, energy efficiency and timing, layer thickness, orientation, as well as debinding and sintering parameters. These elements significantly affect the accuracy of the final product and its mechanical as well as surface properties [11].

The fracture resistance of all ceramic restorations is a significant concern in their clinical use and long-term reliability, influenced by various factors including surface roughness, elastic modulus, crack resistance, and manufacturing techniques [12–14]. Zirconia can inhibit crack propagation by undergoing a transformation from tetragonal to monoclinic structure, which slightly increases the crystal volume and generates beneficial compressive stresses around the crack, a process known as transformation toughening [15].

Previous studies reported that milled monolithic zirconia crowns exhibit fracture resistance that can withstand occlusal loading [16], but there are limited studies that focus on the fracture resistance of 3D printed monolithic zirconia crowns. Limited data are presented on their properties and long-term performance, indicating a need for further research.

Accordingly, the main purpose of the present study was to evaluate the fracture resistance, surface roughness, and microhardness of 3D printed zirconia crowns and compare them to their milled counterparts. The null hypothesis was that there would be no difference in the mentioned properties between zirconia crowns fabricated by subtractive or additive techniques.

## Methods

### Sample size

Sample size was based on 95% confidence level and a power of 80% to detect differences in fracture resistance between milled and printed zirconia [14]. The reported mean (SD) fracture resistance after aging = 1642 (127) and 1224 (263) for milled and printed zirconia, respectively. The calculated mean (SD) difference = 418 (206.5) and 95% confidence interval = 263.5 to 572.5. The minimum sample size was calculated to be 11 specimens per group, increased to 12 to make up for laboratory processing problems. The sample size was calculated using MedCalc Statistical Software version 19.0.5 (MedCalc Software bvba, Belgium; https://www.medcalc.org; 2019).

### Specimen preparation

#### Preparation criteria and epoxy resin dies fabrication for zirconia crowns

A typodont mandibular first molar tooth (Basic study model, KaVo Dental GmbH, Germany) was prepared to accommodate a monolithic zirconia crown. The preparation adhered to the following guidelines: 1.5 mm of occlusal reduction, 1–1.2 mm of axial reduction, an axial taper of 8–10 degrees, and a 1 mm heavy chamfer finish line [17]. Twenty-four impression replicas were taken for the prepared tooth using an addition silicon duplicating material and poured with an epoxy resin material (Kema-poxy 150 3D, CMB, Egypt), with an elastic modulus similar to that of dentin. The definitive epoxy dies were numbered and randomly assigned to two groups.

#### Dies scanning and sample design

All dies were scanned with an extraoral scanner (Medit T710, Medit Corp., South Korea), and a CAD software program (Exocad version 3.0, exocad GmbH, Germany) was employed to digitally design 24 identical full-contour anatomical crowns, incorporating a cement gap of 70 µm that started 1 mm from the finish line [18]. The CAD file of each crown was saved in standard tessellation language (STL) format and sent for both methods of fabrication.

#### Zirconia crowns fabrication

In group I, the STL files were imported into a 5-axis milling machine (CORiTEC 150i, imes-icore, Germany) for subtractive manufacturing using milling discs made of 3 mol% yttria-stabilized zirconia (Nacera Zirconia, Germany), followed by sintering in a zirconia furnace (TABEO-1/M/ZIRKON-100, Mihm-vogt GmbH, Germany) according to the manufacturer’s guidelines.

**Fig. 1:**
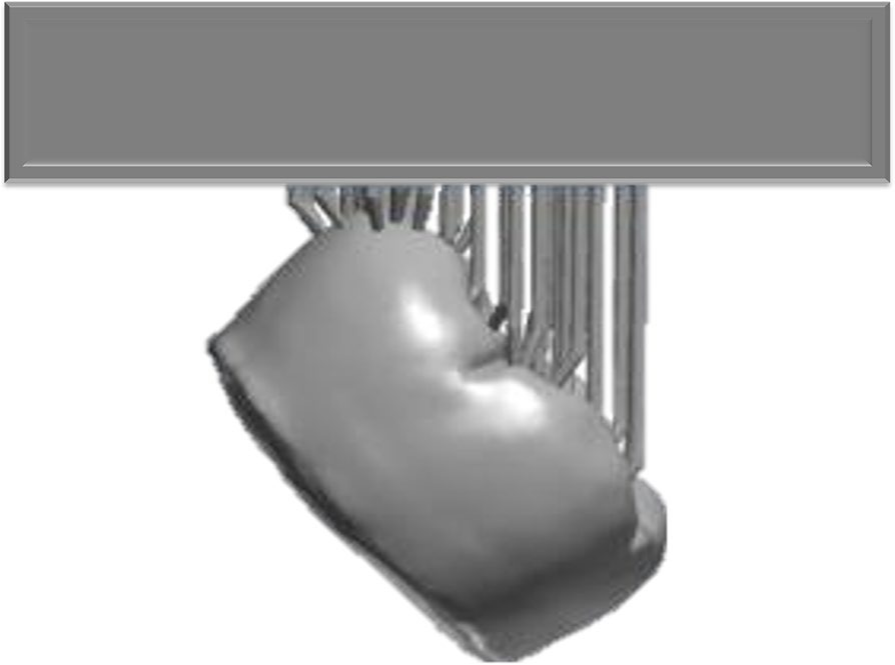
Printing orientation (45°) of 3D printed monolithic zirconia crowns on the building plate

In group II, zirconia crowns were 3D printed using a CeraFab system S65 medical printer (Lithoz GmbH, Austria), which selectively light cure a slurry of 3 mol% yttria-stabilized zirconia particles and an acrylic binding system (LithaCon 210 3y, Lithoz GmbH, Austria) in a layer-by-layer manner. The slurry had a 44.3% solid loading, a density of 3.3 kg/dm^3^, and a dynamic viscosity of 14 Pa.s. The layer thickness used was 25 µm with exposure intensity of 110 mJ/cm^3^ for 36 s/layer and the curing depth was 235 µm. Subsequently, the printed objects were cleaned with the specific solvent (LithaSol 30, Lithoz GmbH, Austria), the resin binder was removed, and then they were sintered to achieve fully dense zirconia restorations. The debinding and sintering were carried out in a single-step process using an LHTC 08/16 special oven (Naberthem GmbH, Germany). The process took about 4 days and reached a maximum temperature of about 1500 °C, which was then held for about two hours. The printing orientation (45°) of one of the zirconia crowns is presented in Fig. 1.

Glazing for both groups was done using Universal overglaze (Dentsply Sirona, Germany) according to manufacturer’s instructions [19].

#### Crowns cementation

**Fig. 2:**
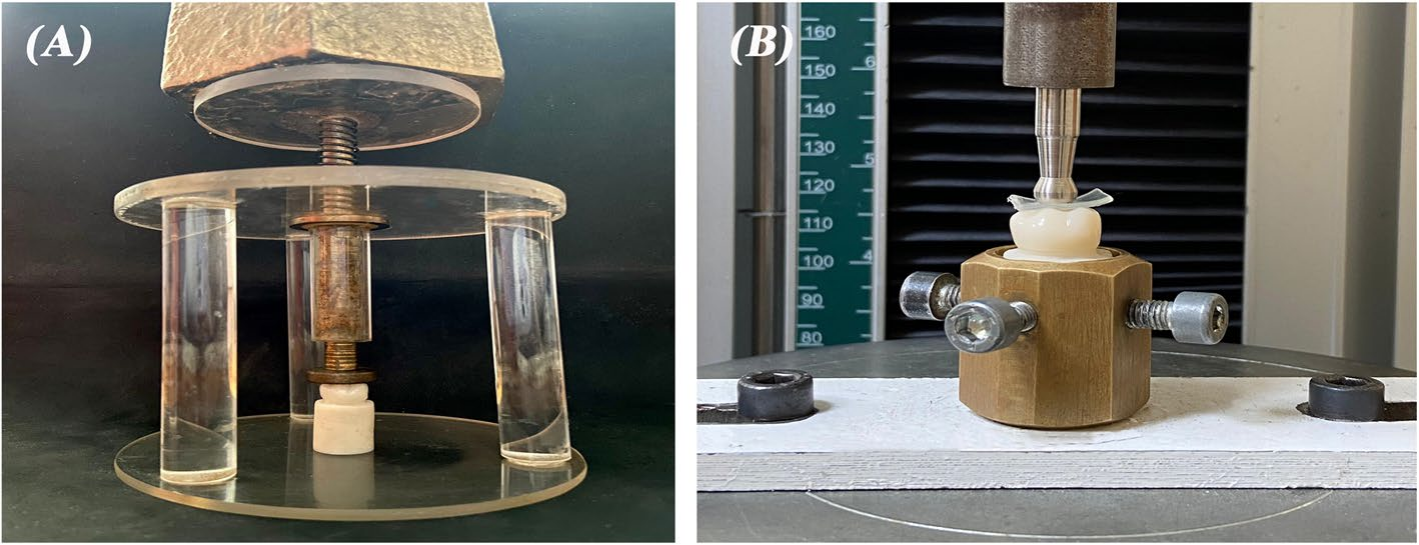
**A** static loading device used during crown cementation. **B** fracture resistance test setup on universal testing machine

A sharp dental explorer was utilized to verify the proper seating of each crown on its respective epoxy resin die prior to cementation. The fitting surface of the crowns was sandblasted with 50 µm alumina particles at a pressure of 2 bar for 10 s from a distance of 10 mm [20]. The crowns were then luted onto the corresponding epoxy dies using a dual cure resin cement (Duo-Link, Bisco, USA) [14]. A static load of 5 kg was applied to provide uniform load to the crowns during cementation (Fig. 2A). Initial light curing was performed for 4 s, followed by the removal of excess cement with a scalpel blade, and then curing for 20 s on each surface to achieve complete polymerization [21].

#### Zirconia discs fabrication

An STL file of a disc 10 mm in diameter and 2 mm in thickness was supplied to the milling machine and 3D printer to fabricate 72 discs of which 36 were milled and the others were printed. All discs were polished using sandpaper from 320–1200 under water cooling for 5 min each then cleaned with ultrasonic cleaner (T-14, L&R manufacturer, USA) [22]. 12 discs of each group were glazed, while 24 were left unglazed. Half of the unglazed discs were used for microhardness assessment and the other half for surface roughness, which was compared to the glazed discs.

#### Thermocycling

All crowns and disc specimens were subjected to 5000 thermal cycles using a customized thermocycling machine for a temperature range of 5–55 °C and a dwell time of 30 s. This setup simulated 6 months of service intraorally [23].

### Fracture resistance, Weibull analysis, and fractographic analysis

The crown specimens were mechanically tested using a universal testing machine (5ST, Tinius Olsen, England). Each specimen was fixed in a specially designed cylindrical copper mold that is attached to the lower plate of the testing machine. A stylus with 6 mm diameter custom made stainless-steel ball was fabricated and installed in the upper arm of the apparatus. A vertical compressive load was applied at the center of the crown along the specimen’s vertical long axis at a crosshead speed of 0.5 mm/min until failure with a 0.5 mm thermoplastic sheet in between to achieve homogenous stress distribution and minimize the transmission of local force peaks (Fig. 2B, C). Fracture resistance was carried out and calculated in Newton (N) with the aid of computer software [21]. Weibull analysis was performed using Origin software (OriginPro, Version 2025, OriginLab Corp., USA) to estimate the Weibull modulus (m) and the characteristic fracture load (σ0) at 95% confidence interval (CI) [24]. Fractographic analysis was performed using scanning electron microscope (SEM) (JSM-IT200, JEOL, USA) after gold sputtering of the specimens to visualize fracture patterns [16].

### Surface roughness

Surface roughness was assessed using a contact stylus profilometer (Marsurf PS10, Mahr, Germany) before and after glazing with a speed of 0.5 mm/s and cut-off length of 0.25 mm. The parameters Ra, Rz, Rp, and Rv were measured in µm [25].

### Vickers microhardness

Vickers microhardness tester (HVS-1000A, Jinan Hensgrand Instrument Co. Ltd., China) was used to apply 500-g force for 10 s to each unglazed disc specimen. The average of 3 readings was used to calculate the microhardness in VHN [24].

### Statistical analysis

Normality was tested using descriptive statistics, Q-Q plots, histograms, and normality tests. All data showed normal distribution, so means and standard deviation (SD) were calculated, and parametric tests were used. Comparisons between the study groups were performed using independent samples t-test, with calculation of mean differences and 95% confidence intervals (CIs). Two-way ANOVA was performed to assess the association between material (milled or printed) and glazing (glazed or unglazed) with surface roughness. Adjusted means and 95% CIs were calculated. Significance level was set at *p*-value < 0.05. Data were analyzed using IBM SPSS for Windows (Version 26.0).

## Results

**Fig. 3:**
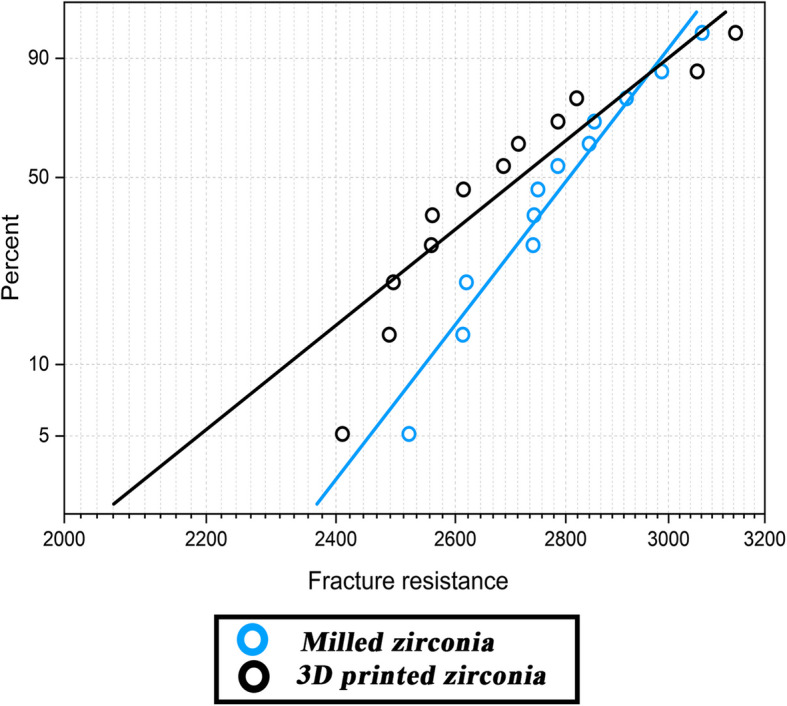
Weibull analysis for milled and 3D printed zirconia after fracture resistance

Milled zirconia showed higher fracture resistance values compared to the printed type (Table 1), however, the difference was not statistically significant (*P* = 0.26). According to Table 2 and Fig. 3, milled zirconia showed higher Weibull modulus (19.5) and characteristic strength (2859.1 N) compared to the printed counterpart (12.1, 2798.6 N).

**Table 1:** Comparison of fracture resistance (N) and microhardness (VHN) between milled and 3D printed zirconia groups

Test	Group	Mean ± SD	Difference (95% CI)	*P-*value
Fracture resistance (N)	Milled	2786.3 ± 160.5	92.6 (−73.1, 258.4)	0.26
	3D printed	2693.6 ± 225.6		
Microhardness (VHN)	Milled	1657.2 ± 32.7	302.1 (27.8, 330.4)	< 0.001^*^
	3D printed	1355.1 ± 34.1		

Independent samples t-test was used

*SD* Standard Deviation, *CI* Confidence Interval

^*^Statistically significant at *P*-value < 0.05

**Table 2:** Weibull estimate for milled and 3D printed zirconia groups

Group	m (Weibull shape)				σ_0_ (Weibull scale)			
	Estimate	SD error	Lower	Upper	Estimate	SD error	Lower	Upper
Milled	19.5	4.2	12.7	29.8	2859.1	45.0	2772.3	2948.6
3D printed	12.1	2.5	8.0	18.2	2798.6	71.2	2662.4	2941.7

*SD* standard deviation

**Fig. 4:**
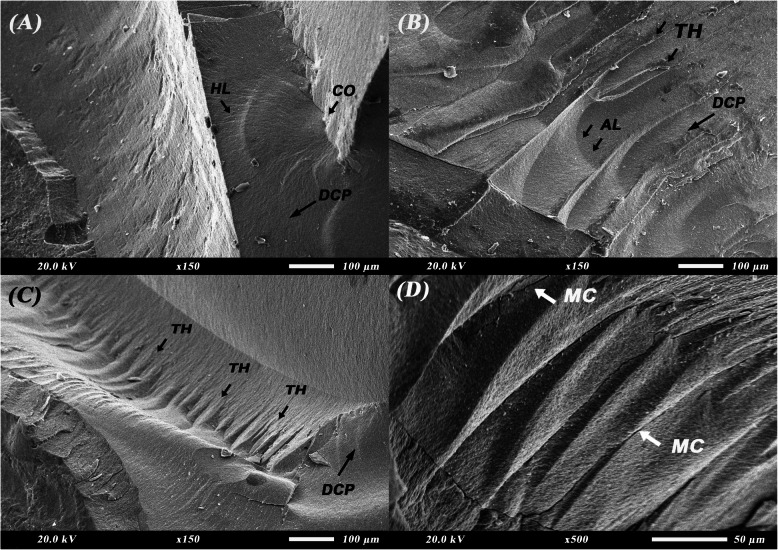
Scanning electron microscope (SEM) images for fractographic analysis of milled zirconia crowns after fracture resistance test. **A** fracture mirror and hackle lines (× 150). **B** twist hackles and arrest lines (× 150). **C** twist hackles (× 150). **D** twist hackles with interpenetrating microcracks (× 500). AL: arrest lines; CO: crack origin; DCP: direction of crack propagation; HL: hackle lines; MC: microcracks; TH: twist hackles

Figure 4 shows the fractographic features upon SEM examination of milled zirconia crowns after fracture. Characteristic features of brittle failure of ceramic can be observed. In Fig. 4A, a fracture mirror can be seen indicating fracture origin along with hackles radiating from it to indicate direction of crack propagation. Twist hackles can be seen in Fig. 4B-D representing the shift in the crack propagation direction. Figure 4B shows multiple arrest lines representing resistance to crack propagation.

**Fig. 5:**
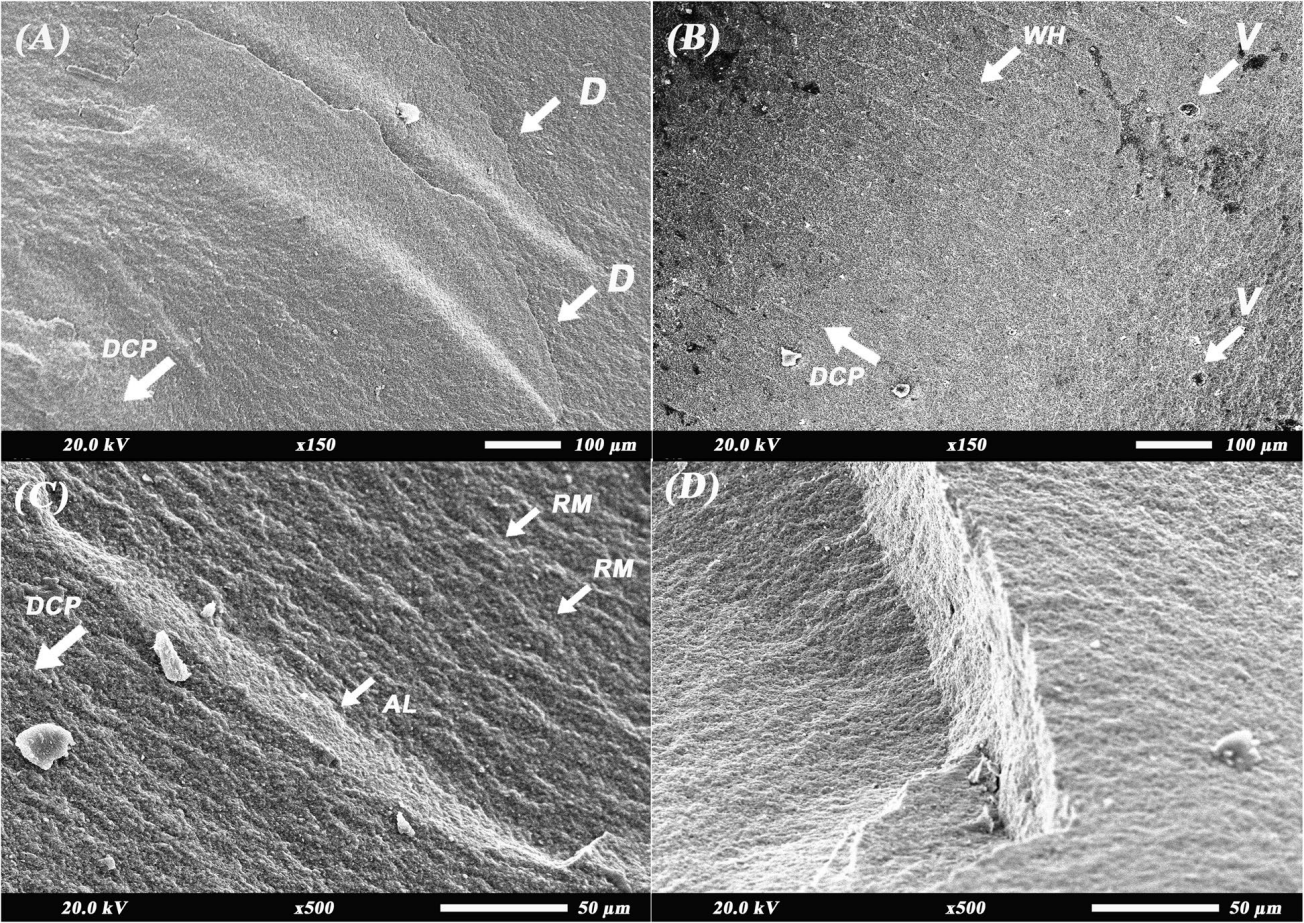
Scanning electron microscope (SEM) images for fractographic analysis of 3D-printed zirconia crowns after fracture resistance test. **A** layer delamination (× 150). **B** wake hackles and voids (× 150). **C** river marks, cleavage, and arrest line (× 500). **D** layer separation at layer line (× 500). AL: arrest lines; D: delamination; DCP: direction of crack propagation; RM: river marks; V: voids; WH: wake hackles

SEM micrographs of the fractographic features of 3D printed zirconia crowns after fracture resistance test are depicted in Fig. 5. The fracture features reflect the effect of the layering technique used during fabrication of the 3D printed specimens on material behavior during failure and fracture. In Fig. 5A and D, delamination and layer separation can be seen. Voids and pores resulting from the debinding process and presence of resin can be observed in Fig. 5B accompanied by wake hackles. Surface irregularities, cleavage, and river marks can be seen in Fig. 5C indicating brittle failure and high-speed propagation of the crack.

Table 3 shows the results for surface roughness of the study groups across various roughness parameters (Ra, Rz, Rp, and Rv). The milled group showed lower roughness values compared to the printed group and glazing had a positive impact by lowering the surface roughness for both groups. There was a statistically significant difference between both groups (*P* < 0.001) for all tested parameters as well as within the same group for the glazed and unglazed subgroups of milled and 3D printed zirconia (Table 3). Both the used material and the glazing as well as the interaction between them had a statistically significant impact on the surface roughness of zirconia (*P* < 0.001, *P* < 0.001, *P* = 0.01) (Table 4).

**Table 3:** Comparison of surface roughness parameters (Ra, Rz, Rp, and Rv) in µm between milled and 3D printed zirconia groups

**Parameter**		**Milled**	**3D printed**	**Difference (95% CI)**	***P-*value 1**
		**Mean ± SD**			
Ra	Glazed	0.23 ± 0.02	0.40 ± 0.05	−0.17 (−0.20, −0.13)	< 0.001^*^
	Unglazed	0.28 ± 0.03	0.52 ± 0.09	−0.24 (−0.30, −0.19)	< 0.001^*^
	*P-*value 2	< 0.001^*^	0.001^*^		
Rz	Glazed	0.75 ± 0.25	1.45 ± 0.24	−0.70 (−0.93, −0.47)	< 0.001^*^
	Unglazed	1.15 ± 9.41	2.39 ± 0.52	−1.24 (−1.68, −0.80)	< 0.001^*^
	*P-*value 2	0.02^*^	< 0.001^*^		
Rp	Glazed	0.45 ± 0.05	0.79 ± 0.20	−0.34 (−0.48, −0.20)	< 0.001^*^
	Unglazed	0.61 ± 0.21	1.25 ± 0.34	−0.64 (−0.91, −0.38)	< 0.001^*^
	*P-*value 2	0.03^*^	0.002^*^		
Rv	Glazed	0.33 ± 0.18	0.68 ± 0.16	−0.35 (−0.51, −0.19)	< 0.001^*^
	Unglazed	0.56 ± 0.25	1.17 ± 0.20	−0.61 (−0.83, −0.40)	< 0.001*
	*P-*value 2	0.03^*^	< 0.001^*^		

*SD* Standard Deviation, *CI* Confidence Interval

*P*-value 1: Comparison between milled and 3D printed using independent samples t-test

*P*-value 2: Comparison between glazed and unglazed using independent samples t-test

^*^Statistically significant at *P*-value < 0.05

**Table 4:** Two-way ANOVA for the association of material and glazing with surface roughness (Ra)

		**Adjusted mean (SE)**	**95% CI**	***P-*value**
Material	Milled	0.25 (0.01)	0.23, 0.28	< 0.001^*^
	3D printed	0.46 (0.01)	0.44, 0.48	
Glazing	Glazed	0.31 (0.01)	0.29, 0.34	< 0.001^*^
	Unglazed	0.40 (0.01)	0.38, 0.42	

Model F: 72.02, *P*-value < 0.001*, Adjusted *R*^2^ = 0.82

*P*-value of interaction material*glazing = 0.01*

*SE* Standard Error, *CI* Confidence Interval

^*^Statistically significant at *P*-value < 0.05

As for the microhardness test, milled zirconia showed a statistically significant higher mean value for microhardness compared to the printed group (*P* < 0.001) (Table 1).

## Discussion

This study discussed the fracture resistance, surface roughness, and microhardness of milled and 3D printed monolithic zirconia used for fabrication of fixed dental prostheses. The null hypothesis was accepted for the fracture resistance test and rejected for the surface roughness and microhardness tests as there was a significant difference between the two fabrication techniques regarding the tested properties.

Milled zirconia showed higher fracture resistance compared to the 3D printed one which could be attributed to the fabrication technique where the zirconia blocks used for milling are more compact and undergo high compaction pressure upon their fabrication leading to lower porosity and smaller internal flaws [26]. However, 3D printed zirconia would suffer from shrinkage and some inherent porosity related to the high viscosity of ceramic slurry and debinding step during the fabrication process (Fig. 4B). Some contraction could also result from the post-curing procedures that occur after printing and before sintering [27] or the difference in thermal expansion coefficient of resin and zirconia [28]. Despite the aging, both groups showed fracture resistance exceeding that of the normal biting forces (450–520 N) [14] and the printed group expressed fracture resistance values that were considerably close to those of the milled ones. According to Kim et al. and Marsico et al., the printing orientation as well as layer thickness play a role in accuracy of the printed structure as well as its bulk and surface properties. The 45° printing orientation used in this work led to better compaction and cohesion of the printed layers offering a higher resistance to the applied forces and the aging procedures [10, 29]. The use of a layer thickness of 25 µm also contributed to more efficient curing of the resin during printing and better cohesion between the zirconia particles during the post-processing steps [29].

The Weibull modulus obtained in our study was higher for milled compared to printed zirconia showing higher predictability of failure, higher reliability, and less scatter of fracture resistance data (Fig. 3). This is also supported by SEM images where the milled zirconia (Fig. 4) is showing evidence of crack propagation resistance through presence of multiple arrest lines and twist hackles, while the 3D printed one (Fig. 5) shows pores, river marks, and layer separation. Despite this, the values for the modulus and characteristic strength of the printed zirconia were considerably higher than those obtained by Kim et al. and Lu et al. [10, 30]. This can be attributed to the difference in printing technique, printing orientation, and layer thickness used.

Surface roughness indicates the material’s ability to resist plaque accumulation and maintain its esthetic appearance [31]. Due to the layering technique used in fabrication of 3D printed crowns, the surface of such specimens exhibits a rough surface as it appears similar to step-stairs which explains the increased roughness of the 3D printed zirconia compared to the milled one [32]. However, the roughness values in this study were considerably lower than those obtained by Patil et al. and Xiang et al. who tested surface roughness of 3D printed zirconia [33, 34]. This suggests that printing using DLP technique, in contrast to SLS or SLA, as well as using a layer thickness of 25 µm and 45° orientation, which were used in this study, had a strong impact on decreasing the roughness values of the printed specimens. After the addition of glaze, there was a significant decrease of roughness in the 3D-printed group as the material occupied the gaps and pores on the zirconia surface [35]. This is also evident through the decrease in the Rv parameter after glazing specifically in the 3D printed group (Table 3).

Rough surfaces create stress concentration points which lead to crack initiation and propagation thus lowering the fracture resistance [30]. The use of glaze with 3D printed crowns in our study could have been one of the contributing factors to binding the surface of 3D printed zirconia and decreasing the stress concentration that results from rough surfaces leading to improved fracture resistance which is comparable to the milled counterpart. Due to surface defects and porosity of the 3D printed zirconia, fluid uptake during thermocycling would be considerably higher leading to low temperature degradation resulting in deterioration of the mechanical properties and increasing the speed of crack propagation [26]. The closure of surface pores due to glazing offered some resistance to the fluid uptake during thermocycling leading to resistance of deterioration of surface properties and fracture resistance after aging.

The presence of defects such as voids, pores, and delamination affects the long-term durability of the final restoration. Excessive porosity near the margins lead to microleakage and secondary caries. With heavy occlusal forces, especially in the posterior regions, fatigue failure and premature wear could originate near areas of voids and delamination. Debonding and impaired restoration retention could also be related to presence of delamination zones. Mitigating these defects through optimizing the printing parameters and the zirconia slurry composition contributes to increasing the survivability of the restoration during function in the oral environment [31, 36].

Surface hardness of materials is considered a measure of their resistance to surface penetration and indentation. Milled zirconia showed superior surface hardness compared to the 3D printed type as a result of the strong bonds and coalescence between the particles due to the high compaction pressure during the fabrication of the milling blocks. On the contrary, 3D printed structures suffer from lack of adhesion between the different layers as well as porosities and voids during their manufacturing resulting in lower surface hardness compared to the milled ones [31]. According to Mei et al., the porosities in DLP printed zirconia are produced due to the high viscosity of the ceramic slurry used during printing and they tend to agglomerate on the surface in different shapes and sizes leading to a decrease in surface hardness compared to milled zirconia [37].

The limitations of this study include the use of only one type of milled zirconia blocks and one type of zirconia printing technique which is DLP. Further studies should investigate different types of 3D zirconia printing techniques and their effect on fracture resistance, surface roughness, and microhardness.

## Conclusions

While 3D-printed zirconia exhibits comparable fracture strength to milled counterparts, limitations in reliability and surface properties highlight the need for further optimization before full clinical adoption.

## Data Availability

The data that support the findings of this study are available from the corresponding author upon reasonable request.

## Abbreviations

Y-TZP: Yttrium-stabilized Tetragonal Zirconia
CAD-CAM: Computer-Aided Design and Computer-Aided Manufacturing
AM: Additive Manufacturing
3D printing: Three-Dimensional Printing
BJ: Binder Jetting
SLA: Stereolithography
SLS: Selective Laser Sintering
DLP: Digital Light Processing
STL: Standard Tessellation Language
N: Newton
CI: Confidence Interval
SEM: Scanning Electron Microscope
VHN: Vickers Hardness Number
SD: Standard Deviation
